# Achieving volatile potassium promoted ammonia synthesis via mechanochemistry

**DOI:** 10.1038/s41467-023-38050-2

**Published:** 2023-04-22

**Authors:** Jong-Hoon Kim, Tian-Yi Dai, Mihyun Yang, Jeong-Min Seo, Jae Seong Lee, Do Hyung Kweon, Xing-You Lang, Kyuwook Ihm, Tae Joo Shin, Gao-Feng Han, Qing Jiang, Jong-Beom Baek

**Affiliations:** 1 School of Energy and Chemical Engineering/Center for Dimension-Controllable Organic Frameworks, Ulsan National Institute of Science and Technology (UNIST), Ulsan, South Korea; 2 Key Laboratory of Automobile Materials (Jilin University), Ministry of Education, and School of Materials Science and Engineering, Jilin University, Changchun, P. R. China; 3 Pohang Accelerator Laboratory, Pohang University of Science and Technology, Pohang, South Korea; 4 Graduate School of Semiconductor Materials and Devices Engineering, Ulsan National Institute of Science and Technology (UNIST), Ulsan, South Korea

**Keywords:** Catalyst synthesis, Chemical engineering, Porous materials

## Abstract

Potassium oxide (K_2_O) is used as a promotor in industrial ammonia synthesis, although metallic potassium (K) is better in theory. The reason K_2_O is used is because metallic K, which volatilizes around 400 °C, separates from the catalyst in the harsh ammonia synthesis conditions of the Haber-Bosch process. To maximize the efficiency of ammonia synthesis, using metallic K with low temperature reaction below 400 °C is prerequisite. Here, we synthesize ammonia using metallic K and Fe as a catalyst via mechanochemical process near ambient conditions (45 °C, 1 bar). The final ammonia concentration reaches as high as 94.5 vol%, which was extraordinarily higher than that of the Haber-Bosch process (25.0 vol%, 450 °C, 200 bar) and our previous work (82.5 vol%, 45 °C, 1 bar).

## Abstract (web summary)

Potassium oxide is used as a promotor in industrial ammonia synthesis, although metallic potassium is better in theory. Here, the authors demonstrate metallic potassium, an unstable metal that easily volatilizes at high temperature, can be used as a promotor for ammonia synthesis.

## Introduction

Ammonia (NH_3_) has the highest nitrogen (N) content of any commercial fertilizer, and its use has largely freed the world from famine for over one hundred years; it is the second most produced chemical worldwide^1,2^. Recently, NH_3_ has attracted interest as a potential hydrogen (H) carrier to help realize net-zero emissions in the green hydrogen economy^3–6^. Developing an efficient catalyst for ammonia synthesis is one of the major challenges for several reasons. The exothermic nature of ammonia synthesis (N_2_ + 3H_2_ ↔ 2NH_3_, − 92.44 kJ mol^−1^) favors low temperature, but dissociating stable diatomic triple-bonded nitrogen (N≡N) requires high temperature^7–12^, which results in a sluggish overall reaction rate and low ammonia concentration^13,14^. To speed up ammonia synthesis, in industry, potassium oxide (K_2_O) is frequently used as a promotor^7,15^, which can accelerate N_2_ chemisorption and dissociation on the surface of iron (Fe) catalysts^16^. This is because the potassium (K) in the K_2_O, as one of group 1 A metals, can easily donate electrons to Fe and N_2_ (refs. ^3,17–19^). Consequently, the K, as an electronic promotor, increases the sticking coefficient of N_2_ to Fe, weakens the bond strength of N_2_, and lowers the activation energy of N_2_ dissociation^16,18,20–22^.

**Fig. 1:**
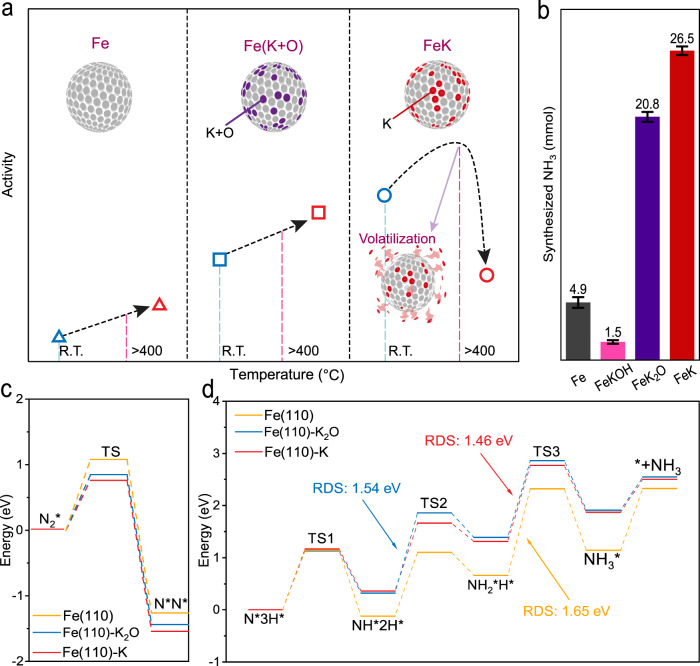
Comparison of Fe catalytic activity with different K promotors. **a** Schematic of Fe catalytic activity with different promotors depending on reaction temperature. Pure K and oxygenated K derivatives (K_2_O and KOH, both denoted as K + O) are used as K promotors for NH_3_ synthesis. At a temperature above 400 °C, the activity of K on Fe (FeK) is diminished by the K desorption from the surface of Fe catalyst. Therefore, oxygenated K derivatives on Fe [Fe(K + O)s] show better performance above 400 °C. However, at a temperature below 400 °C, the K can stably stick to the surface of FeK, showing much higher activity than Fe(K + O)s. Color code in the cartoon: gray, dark red, and purple circles are Fe, K + O, and K, respectively. **b** Comparison of synthesized NH_3_ concentration with same catalyst weight, using different K promotors (K, K_2_O, and KOH) at the same conditions [same loading ratio of K to Fe (2.5 at%), 45 °C] via mechanochemical process. For N_2_ dissociation step, ball-milled for 11.5 h after charging N_2_ (9 bar). For hydrogenation step, ball-milled for 3 h after charging H_2_ (9 bar). The error bars are determined from at least five independent experiments. Density functional theory calculations of mechanochemical ammonia synthesis with different catalytic systems (FeK, FeK_2_O, and Fe): **c** nitrogen dissociation step; **d** hydrogenation step.

However, the oxygen (O) in K_2_O disturbs Fe, to chemisorb N_2_ (refs. ^23–25^). Therefore, a Fe catalyst with pure metallic K would work better for ammonia synthesis^23,25,26^. Unfortunately, however, pure metallic K is not thermally stable. It is prone to evaporate from the surface of the Fe catalyst at around 400 °C. As a result, compared to K_2_O (740 °C), pure metallic K (melting at 63.5 °C) cannot be used as a promotor under the Haber–Bosch reaction conditions (450 °C, Fig. 1a). That is to say, using high-temperature-stable K_2_O as a promotor in ammonia synthesis provides a compromise solution^23,26^. To maximize the use of K as a promotor without oxygen poisoning, ammonia synthesis must be conducted below 400 °C.

In the previous work, we realized ammonia synthesis in mild conditions (at 45 °C, 1 bar) via mechanochemical ball-milling Fe powders and sequentially injecting N_2_ and H_2_. In the present study we focused on improving the performance of the Fe catalyst with a pure metallic K promotor (FeK). As a result, the rate of weighted NH_3_ synthesis (mmol kWh^−1^) was approximately 5 times higher compared to the previous work (using pure Fe catalyst without K promotor in the same conditions). Furthermore, the minimum rotation speed for hydrogenation was only 100 r.p.m., which was considerably lower than using pure Fe (350 r.p.m.). We found a new opportunity to use pure metallic K as a promotor to boost the efficiency of ammonia synthesis for commercial production without the risk of oxygen poisoning of the Fe catalyst.

Iron (Fe), which is one of the most earth-abundant elements, is exclusively used as the key catalyst in conventional ammonia synthesis via the Haber-Bosch process because of its activity with high economic benefit^27,28^. We previously realized ammonia synthesis using a Fe catalyst near ambient conditions (at 45 °C, 1 bar) for the first time, via mechanochemical process^29^. However, for commercial-scale production, two issues should be resolved. One is bringing ‘down’ the mechanochemical energy (rotation speed) needed to initiate nitrogen (N_2_) dissociation and subsequent hydrogenation into ammonia. The other is bringing ‘up’ ammonia concentration for higher throughput. If we can find an efficient promotor that works under milder conditions, those two issues can be simultaneously resolved for both large-scale production and high productivity at a low-cost.

## Results and discussion

### Different potassium-based promotor studies

Potassium oxide (K_2_O) is the most frequently used promotor in the state-of-the-art Haber-Bosch process^7^. It provides us tantalizing clues about how to tackle these issues. For comparison, pure metallic K (without O) and its oxygenated derivatives (K_2_O, and KOH, both denoted as K + O) were chosen as promotors under the same mechanochemical reaction conditions (at 45 °C, 1 bar, Fig. 1a). The results showed that both K and K_2_O had better activity than pure Fe (Fig. 1b and Supplementary Fig. 1). On the other hand, KOH performance was inferior to the pure Fe, because KOH induced severe agglomeration of the Fe catalyst, and deactivation. Density functional theory (DFT) calculations demonstrate that the activity of both nitrogen dissociation and hydrogenation steps follow the sequence of FeK > FeK_2_O > metallic Fe (Fig. 1c, d and Supplementary Fig. 2), which matches well with our experiment results (Fig. 1b). The K promotes Fe via electronic effect, and O weakens the promotion effect of K to a certain extent. As anticipated, pure K was the best promotor among them.

Next, the relationship between overall catalytic activity and K loading amount was investigated. When the loading amount of K promotor relative to Fe catalyst was increased up to 5.0 at%, ammonia yield increased (Supplementary Fig. 3). However, a loading amount higher than 2.5 at% resulted in agglomeration of the Fe, deteriorating catalytic performance over repeated experiments (Supplementary Fig. 4). Based on this result, we concluded that the optimum K loading was ~2.5 at%. All further experiments were carried out with a K loading of 2.5 at%, unless specified.

Before we investigated the kinetic studies of ammonia synthesis, we optimized the ball size and ball type for the system. First, we compared the activity of nitrogenation and ammonia synthesis for three different ball sizes (hardened steel balls with diameters of 3, 5, 10 mm, Supplementary Fig. 5) at the same rotation speed. The correlation between ball size and activity indicated that balls with 5 mm diameter were the optimum ball dimension, because there is a trade-off between collision frequency and single-ball kinetic energy. In addition, we tested the zirconium oxide (ZrO_2_) balls (5 mm) to compare with steel balls (5 mm, Supplementary Fig. 6). Because the ZrO_2_’s mass density is lower than Fe, we used the same number of ZrO_2_ balls and calculated the rotation speed required to maintain the same kinetic energy as the Fe balls. We found that ZrO_2_ balls were a bit inferior to Fe balls, which can also provide catalytically active surfaces of Fe balls, while the ZrO_2_ balls do not.

### Ammonia synthesis kinetics studies

**Fig. 2:**
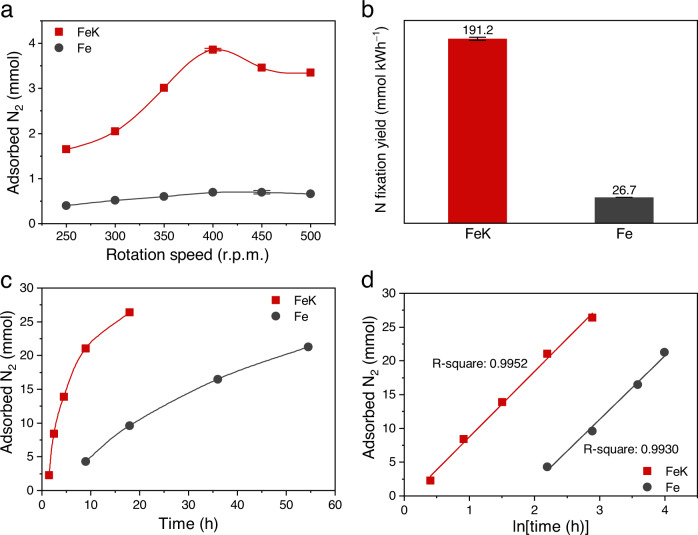
N_2_ dissociation kinetics. **a** The amount of adsorbed N_2_ with respect to rotation speed. The total rotating cycles for FeK were 108,000 cycles, and for pure Fe were 240,000 cycles. Data were normalized to 30,000 cycles (1 hour at 500 r.p.m.). The data of Fe were adapted from ref. ^29^. **b** Highest N* fixation activity comparison between FeK and Fe. N* fixation yield was normalized by consumed energy (kWh) (FeK 400 r.p.m., Fe 450 r.p.m.). The data of Fe were adapted from ref. ^29^. The error bars (Fig. 2a, b) are determined from at least five independent experiments. **c** The amount of adsorbed N_2_ with respect to ball-milling time. The rotation speed was 400 r.p.m. for both FeK and pure Fe. **d** The amount of adsorbed N_2_ and the natural logarithm of the ball-milling time [ln(time)] are linearly dependent. Incubation period (FeK, 1.1 h; pure Fe, 6.0 h) could be determined by extrapolation to the origin. The weight of FeK and Fe was 23.8 g and 24 g.

Reaction kinetics were then studied. First, we tried to identify the optimum rotation speed for N_2_ dissociation. The relationship between catalytic activity and the rotation speed showed a volcano-type dependence. The highest amount of adsorbed N_2_ was obtained at 400 r.p.m. (Fig. 2a). This result suggests that as the mechanical energy increases, it helps N_2_ dissociation in the early stage, and then hinders the reaction progress. This is because the heat generated by the mechanical friction facilitates the recombination of dissociated N (N*) atoms into N_2_ (ref. ^7^). To compare between the highest nitrogen fixation activity of each sample, the data were further normalized by consumed energy to eliminate the effect associated with the different rotation speeds (Fig. 2b). The nitrogen fixation yield normalized by consumed energy of FeK (400 r.p.m.) was 7 times higher than that of Fe (450 r.p.m.)^29^.

We then conducted an experiment to determine the amounts of adsorbed N_2_ as a function of ball-milling time at a fixed rotation speed of 400 r.p.m. (Fig. 2c). The K-promoted Fe catalyst (FeK, 14.5 h) required a much shorter time to adsorb the same amount of N_2_ (25 mmol) as the pure Fe catalyst (Fe, 83.3 h). The K boosted the turnover frequency of Fe, because it donates electrons to the Fe and N_2_ (ref. ^26^). From data fitting, we found that the natural logarithm of ball-milling time [ln(time)] and the amount of adsorbed N_2_ have a linear relationship (Fig. 2d). The fitted lines imply that there should be an incubation period for N_2_ dissociation. We used an extrapolation method to determine the incubation period, which was approximately 1.1 h for FeK, and 6.0 h for pure Fe. The FeK catalyst can trigger N_2_ dissociation in a much shorter time than the pure Fe catalyst, because the K helps pulverize and activate the raw Fe powders.

**Fig. 3:**
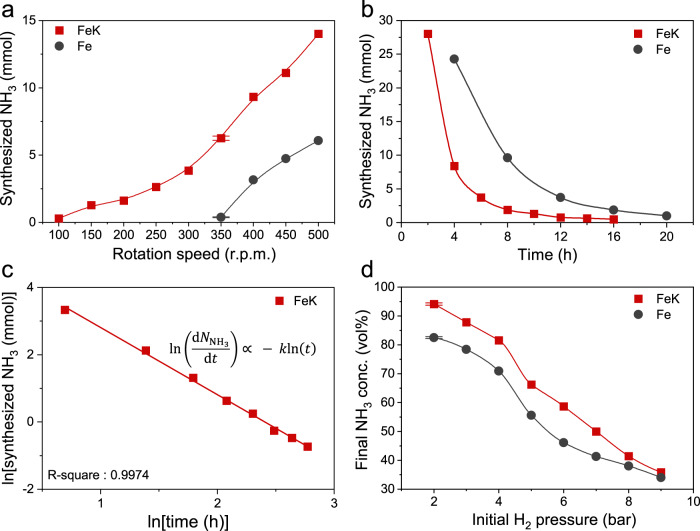
Ammonia yield kinetics. **a** The amount of synthesized NH_3_ versus rotation speed. The N_2_ absorbed FeK powders were prepared by ball-milling in N_2_ pressure (9 bar) for 18 h. The total number of rotation cycles for FeK were 60,000, and for N_2_ absorbed Fe were 120,000. Data were normalized to 30,000 cycles (at 500 r.p.m. for 1 h). **b** The amount of synthesized NH_3_ versus time, which is the hydrogenation rate at the rotation speed of 500 r.p.m. for both FeK and Fe. Hydrogenation was repeated after charging H_2_ pressure (9 bar each) every 2 h for FeK, and every 4 h for Fe. **c** Relation between the natural logarithm of the synthesized NH_3_ *vs*. the natural logarithm of time shows a linear dependence for FeK. **d** Final NH_3_ concentration with respect to initially charged H_2_ pressure. Each sample was ball-milled for hydrogenation for 2 h and 4 h after charging 9 bar of H_2_. The weight of FeK and Fe was 23.8 g and 24 g. All the data of Fe were adapted from ref. ^29^. The error bars (Fig. 3a, d) are determined from at least five independent experiments.

Unlike the N_2_ dissociation step, the hydrogenation step to ammonia increased (Fig. 3a) as rotation speed was raised. This is associated with endothermic hydrogenation, which has a higher equilibrium constant at a higher temperature^7^. Therefore, the increased mechanical energy accelerates hydrogenation. The activity of FeK at 350 r.p.m. was over 15 times higher than that of the pure Fe catalyst. Most importantly, the minimum required rotation speed to initiate hydrogenation was only 100 r.p.m. for FeK, which was much lower than the pure Fe (350 r.p.m., Fig. 3a). The activity curves for FeK (non-monotonic derivative) and Fe (monotonic derivative) featured different shapes, because K alters the reaction equilibrium constant^25^.

One hydrogen charging/ball-milling cycle was insufficient to complete the hydrogenation of all adsorbed N (N*) on FeK into NH_3_. We conducted 9 hydrogen charging/ball-milling cycles (each cycle charged 9 bar) to study hydrogenation kinetics. The rate of hydrogenation exponentially decreased as the cycle number increased (Fig. 3b). This is because the concentration of N* was diluted as the reaction progressed. The numerical analysis as a function of the amount of synthesized ammonia and ball-milling time indicates that there is a linear dependence between the natural logarithm of the amount of synthesized NH_3_ [ln(synthesized NH_3_)] and the natural logarithm of time [ln(time)] (Fig. 3c). This result is totally different than the one using pure Fe, where the linear dependence was between ln(synthesized NH_3_) and time (Supplementary Fig. 7). Density functional theory (DFT) calculations indicated that the different catalysts (FeK and Fe) showed the different rate-determining steps (RDS) for hydrogenation (Fig. 1d). Energy barrier and energy difference between initial state and final state for each step was extremely different due to influence of K. Furthermore, for the hydrogenation kinetics, we studied on the reaction rate constant (*k*) as a function of temperature, and the turnover frequency (TOF) as a function of hydrogen partial pressure and temperature (Supplementary Notes and Supplementary Figs. 8 and 9). The both calculated *k* and TOF results indicated the distinct difference between FeK and Fe. These results further verify that there are different kinetics between FeK and pure Fe catalysts for the hydrogenation step.

The final ammonia concentration depends on the initial charged pressure of H_2_ (Fig. 3d and Supplementary Fig. 10). The final ammonia concentration using FeK could reach up to 94.5 vol% with the initially charged H_2_ pressure (2 bar). The ammonia concentration was much higher than using pure Fe (82.5 vol%)^29^ and the Harbor-Bosch process (25.0 vol%)^7^. After hydrogenation with the initially charged H_2_ pressure (2 bar), the pressure reduced to approximately 1.3 bar, which suggested the hydrogenation could be conducted near atmospheric pressure.

To directly describe the ammonia production rate, a weighted ammonia production rate was calculated (Supplementary Fig. 11 and Supplementary Tables 1 and 2). The highest activity rotation speed for each catalyst was chosen for N_2_ dissociation and hydrogenation. The weighted ammonia production of FeK per time unit (mmol h^−1^) was about quadruple that of pure Fe. To eliminate the effect associated with the different rotation speeds, the data were further normalized by consumed energy. The weighted ammonia production per energy unit (mmol kWh^−1^) was 5-fold higher than pure Fe.

### Theoretical studies

**Fig. 4:**
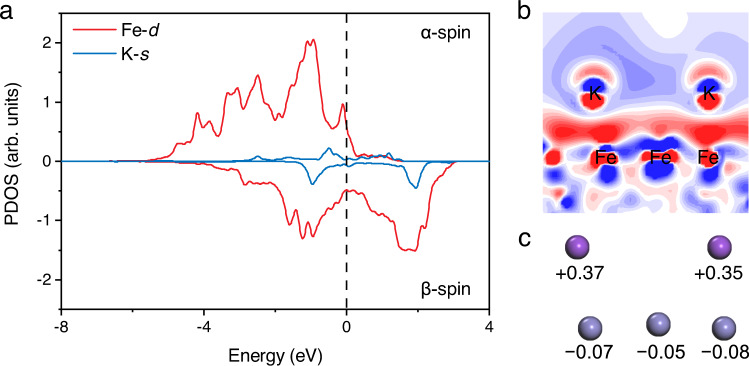
Theoretical study on the electronic effect between Fe and K. **a** Partial density of states (PDOS) of Fe(110)-K, including the Fe-*d* orbits and adjacent K-*s* orbits. **b** Corresponding charge density difference between Fe and K. **c** the Hirshfeld charge analysis. Color codes in the cartoon: purple and gray are K and Fe, respectively. From the Hirshfeld charge analysis in **b**, the red regions represent electron accumulation and the blue regions represent electron depletion, respectively (Max Isovalue: 0.02 e Å^−^^3^).

To rationalize our experimental performance difference between FeK and Fe, the theoretical analysis has been used (Fig. 4). We used partial density of states (PDOS) to study about donating electrons between Fe and K (Fig. 4a). The PDOS of Fe(110)-K, which is projected on the *d* orbitals of Fe atoms and the *s* orbitals of its neighboring K atoms. There are significant hybridizations among Fe-*d* and K-*s* orbitals, indicating that there is a chemical interaction between Fe and K rather than a weak physical adsorption of K on Fe. The electron redistributions in the interface of Fe and K indicate that the electron accumulation occurs to Fe atoms, and the electron depletion arises to K atoms (Fig. 4b). As well, the Hirshfeld charge analysis shows that the K atoms have lost electrons and the nearby Fe atoms are getting electrons (Fig. 4c). Above evidence explain why the performance of FeK has been greatly improved over that of Fe.

### Catalyst analysis

**Fig. 5:**
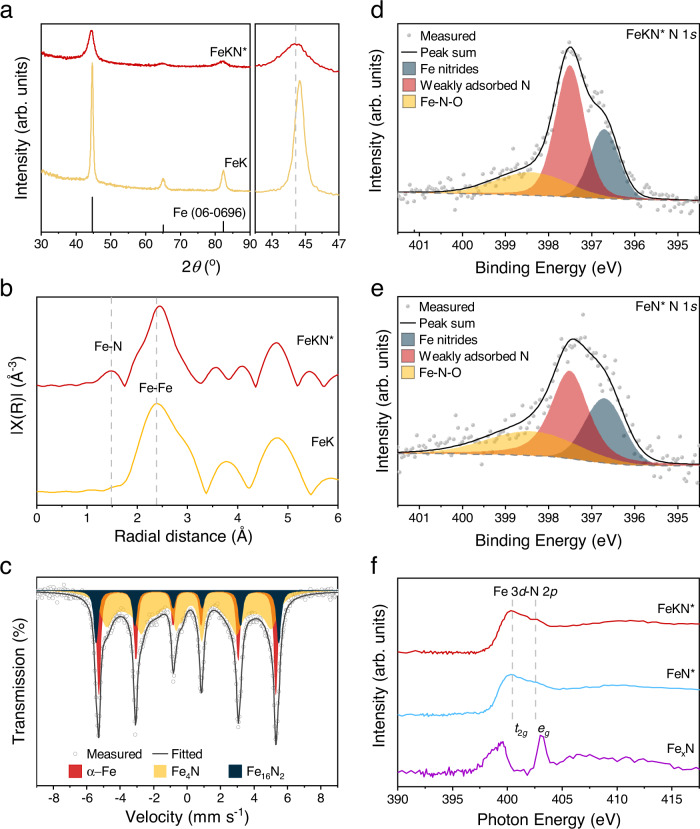
Characterizations of the catalysts. **a** XRD measurements of FeKN*, and regenerated FeK after hydrogenation. **b** Radial distribution function of FeKN* and regenerated FeK after hydrogenation. Radial distribution function was fitted by FeK-edge EXAFS data. **c** Mössbauer spectra of FeKN*. High-resolution XPS N 1 *s* spectra: **d** FeKN* and **e** FeN*. **f** NEXAFS of FeKN*, FeN*, and commercial Fe_x_N (*x* = 2–4).

To analyze their underlying physical nature, the catalysts were characterized. X-ray diffraction (XRD) was used first, and the XRD pattern of the nitrogenated FeK catalyst (named as FeKN*) was found to have shifted to a low angle (Fig. 5a). That is, the lattice parameter had expanded according to Braggs’ law. The result indicates that the N* atoms had diffused into the bulk inside and were located at interstitial positions. This verified the fact that the dissociative N* atoms were not stationary, and instead had high mobility. The high mobility of the adsorbed N* atoms is good for the catalytic reaction, because it can provide diverse active sites for efficient N_2_ dissociation and hydrogenation into ammonia. After complete hydrogenation of the FeKN*, the FeK catalyst was regenerated. As we can see, the facets of the regenerated FeK display angles similar to the standard pure Fe powder (PDF No. 06-0696), which means the N* atoms can be thoroughly removed and the FeK can be recycled.

The FeKN* (2.69°) showed a broader full width at half maximum in comparison with its precursor, FeK (0.77°), which means that the FeKN* has much a smaller grain size (Fig. 5a). This kind of phenomenon is very advantageous for ammonia synthesis, because the smaller the grain size after nitrogenation (FeKN*, 3.16 nm), the more surface area the FeKN* can provide to uptake more nitrogen. Returning back to the bigger grain size of FeK (11.20 nm) after the complete hydrogenation of FeKN* into ammonia will be beneficial, by creating more fresh active sites through crushing again. These fresh active sites are available for the subsequent nitrogenation of FeK into FeKN* for recyclability.

Comparing the FeKN* catalyst (3.16 nm) with FeN* (2.56 nm for FeN*, Supplementary Fig. 12), the addition of K had nearly no influence on grain size. This could be associated with the pinning effect of N*, which plays a key role in suppressing grain growth^30^. After complete N* removal via hydrogenation, the grain size of the regenerated FeK (11.20 nm) was much larger than the Fe (7.75 nm), which demonstrates K facilitated the mass transport, promoting N* to diffuse between different sites.

Extended X-ray absorption fine structure (EXAFS) spectroscopy, which can identify the bonding information of neighboring atoms^29^, was then carried out. The radial distribution function derived from EXAFS showed that the FeN bonding formed near 1.4 Å for both FeKN* and FeN* (Fig. 5b, Supplementary Fig. 13). The existence of FeN bonding demonstrates N_2_ dissociation was successful. Furthermore, the Fe-Fe bonding became longer after N_2_ adsorption on the FeK (FeKN*) or Fe (FeN*). These results were quite well matched with the XRD data. The disappearance of FeN bonding in both the regenerated FeK and Fe after the complete hydrogenation of FeKN* or FeN*, respectively, further verifies the adsorbed N atoms can be completely hydrogenated into ammonia.

Mössbauer spectroscopy is an ultra-sensitive technique for characterizing Fe. It can be applied to study a short-range ordered structure. Our analysis determined that FeKN* contained the nearly amorphous Fe_4_N phase (59%) with broad peaks, crystalline Fe_16_N_2_ (15%), and crystalline α-Fe (26%) with sharp peaks (Fig. 5c). This result is quite similar to that of FeN* (Supplementary Fig. 14). With the theoretical analysis, between Fe_4_N and Fe_16_N_2_, the former is expected to show higher hydrogenation performance by comparing RDS step of each structure (Supplementary Fig. 15). By adding K as a promotor, we could have synthesized Fe_4_N with much less time. The Mössbauer spectra of the regenerated FeK (Supplementary Figs. 16 and 17) showed only α-Fe peaks, and this implies that all the N* atoms were converted into ammonia. Once again, the results agreed well with both XRD and EXAFS analyses.

In order to investigate the catalysts’ chemical information, we used X-ray photoelectron spectroscopy (XPS, Fig. 5d, e) and near-edge X-ray absorption fine structure (Fig. 5f). Because the surfaces of metallic K and Fe are extremely sensitive to oxidation while sampling and transferring (Supplementary Figs. 18 and 19), we only focused on the nitrogenated sample (FeKN*). This is because nitrides can hinder oxidation, allowing N 1 *s* analysis. The results indicated that the FeKN* has a higher ratio of weakly bonded FeN (397.5 eV) to strongly bonded FeN (396.6 eV) than FeN*. Although K can theoretically increase the binding energy between Fe and N*, the percentage surface coverage of weakly adsorbed N* in FeK catalyst can be higher than on Fe catalyst. It is because the more active FeK requires a shorter ball-milling time to reach a similar N* content on Fe, resulting in the population of N* on the surface of FeK is higher than inside. The highest binding energy peak (399.0 eV) was assignable to the oxynitrides which seem to be formed during sampling and transferring^31,32^. The N K-edge feature of FeKN* near 401 eV (Fig. 5f) indicates that most of the N* atoms are weakly adsorbed on its surface, rather than in its bulk^33^. A careful inspection indicated the FeKN* shifts to a bit higher photon energy, which matches well with the XPS results (Fig. 5d, e). In addition, the Fe L-edge (Supplementary Fig. 20) indicates there were an evident difference that was not observable between the nitrogenated sample (FeKN*) and its precursor (FeK), because of the low resolution. However, both had lower than commercial iron nitride [Fe_x_N (x = 2–4)]. This result further verifies that the bonding between Fe and dissociative N* is weak. K can weaken the N adsorption, and thus subsequently enhance the ammonia desorption.

## Methods

### FeK catalyst optimization

N_2_ dissociation using iron (Fe) catalyst with potassium (K) as a promoter was performed with the ball-milling method. The experiment was carried out using a planetary ball-milling machine (Pulverisette 6, Fritsch). In an argon (Ar) (99.999%, KOSEM Corp.) atmosphere glove box (UNIlab pro, MBRAUN), iron powder [23.4 g, iron sponge, ~100 mesh, 99.9% (metals basis), Alfa Aesar], potassium (0.420 g, potassium, chunks, in mineral oil, 98%, Alfa Aesar), and steel balls (500 g, Ø = 5 mm, hardened steel with 99 wt% Fe) were charged in a planetary ball-milling jar (500 ml). The volume of the ball-milling jar after placing all materials (Fe, K, and steel balls) was 249 ml. The volume was determined using an extrapolation method with charged nitrogen (N_2_, 99.999%, KOSEM Corp.) volume after injecting 2, 3, 4, 5, 6, 7, 8, and 9 bar of N_2_ gas. After loading all materials in the glove box, N_2_ gas was charged/discharged using a vacuum pump to completely remove the Ar. Then, N_2_ gas (9 bar) was charged into the ball-milling jar.

Ball-milling was conducted in cycles with 30 min run/10 min break manage the heat in the ball-milling jar. The experiment for optimum rotation speed for N_2_ adsorption was conducted by 250, 300, 350, 400, 450, or 500 r.p.m. for 108,000 cycles for each cycle. The experiment to determine the amount of adsorbed N_2_ was conducted at the milling speed of 400 r.p.m. for 1.5, 2.5, 4.5, 9, and 18 h for each. In addition, to prepare the nitrogen-doped catalyst (FeKN*) for hydrogenation, the experiment was conducted at 400 r.p.m. for 18 h.

### Performance comparison of potassium derivatives

The calculated weight of K, K_2_O [made by mixing KO_2_ (potassium dioxide, powder, Sigma-Aldrich) and K in a 1:3 molar ratio because there is no commercial K_2_O], and KOH (potassium hydroxide, anhydrous, ≥99.95% trace metals basis, Sigma-Aldrich) for the same atomic ratio of K (2.5 at%, K/(Fe+K) × 100) with Fe (18 g), and 500 g of steel balls were loaded into the ball-mill jar in the Ar atmosphere glove box. After placing all the materials in the glove box, charging/discharging with N_2_ gas was performed to completely remove the Ar gas using a vacuum pump. After that, we refilled N_2_ (9 bar) into the jar, and each sample was ball-milled at 450 r.p.m. for 11.5 h. These experiments were independently conducted at least 5 times to determine the error bars.

### Different ball sizes and types of balls

Fixed amounts of K (2.5 at%) and Fe powders (18.0 g) were used for these experiments. To generate the same kinetic energy, the same weight (500 g) of different-sized balls (Ø = 3, 5, and 10 mm) were used for each experiment. Furthermore, the rotation speed and time were, respectively, fixed at 450 r.p.m. for 11.5 h after charging nitrogen (9 bar).

For comparison experiments with ZrO_2_ balls, the same size (Ø = 5 mm) and number (423 g) of ZrO_2_ balls were used. Because the ZrO_2_’s mass density is lower than Fe, we calculated the rotation speed (490 r.p.m.) required to maintain the same kinetic energy (${E}_{{{{{{\rm{K}}}}}}}=\frac{1}{2}m\times {v}^{2}$) as the Fe balls to exclude other parameters (energy, time, and so on), while maintaining the same rotation time (11.5 h) and N_2_ pressure (9 bar).

### Hydrogenation for FeK catalyst optimization

Every hydrogenation step experiment was carried out after N_2_ dissociation into FeKN* with ball-milling at 400 r.p.m. for 18 h. Hydrogen (H_2_, 9 bar, 99.999%, Daesung Industrial Gases Co.) was charged into the ball-mill jar after removing remnant N_2_ by a vacuum pump. Ball-milling was conducted in cycle with 30 min run/10 min break to prevent overheating of the ball-milling machine. Each experiment to determine the amount of synthesized NH_3_ depending on the rotation speed was conducted at 100, 150, 200, 250, 300, 350, 400, 450, or 500 r.p.m. for 60,000 cycles. Gas chromatography (GC, Agilent 7890B) was used to determine the gas ratio [NH_3_/(NH_3_ + H_2_)] to calculate the amount of NH_3_. The experiment to analyze the amount of synthesized NH_3_ depending on time was conducted after 8 times of charging H_2_ (9 bar) every 2 h. After each ball-milling, the amount of synthesized NH_3_ with respect to milling time was determined by the gas ratio. Each experiment to determine the final NH_3_ concentration depending on initial H_2_ pressure (2, 3, 4, 5, 6, 7, 8, or 9 bar) was conducted at 500 r.p.m. After ball-milling, GC was used to determine the final concentration and synthesized amount of NH_3_.

### Hydrogenation for others

After the N_2_ dissociation step for each experiment, hydrogen (9 bar) was charged into the ball-mill jar after removing remnant N_2_ by vacuum pump. Each ball-milling was conducted at 500 r.p.m. (540 r.p.m. for ZrO_2_ to maintain the same total kinetic energy as the hardened steel balls) for 3 h.

### FeKN* for air-sensitive analysis

Steel balls (500 g), Fe powder (23.4 g), and K (0.420 g) were loaded in the planetary ball-milling jar in the glove box under Ar atmosphere. After sealing the jar, the Ar gas was removed by vacuum pump and then N_2_ (9 bar) was charged through gas inlet and ball-milled at 400 r.p.m. for 18 h. The sample (FeKN*) was collected in the glove box under Ar atmosphere to prevent possible oxidation, and used for analysis.

### FeK for air-sensitive analysis

After preparing FeKN*, the remnant N_2_ in the ball-milling jar was pumped out using a vacuum pump. Then, hydrogen (9 bar) was charged and ball-milled at 500 r.p.m. This hydrogenation step was repeated a few times until no NH_3_ was detected by GC. After the hydrogenation was completed, the sample (FeK) was collected in the glove box under Ar atmosphere to prevent possible oxidation, and used for analysis.

### Characterizations

XRD was measured using D/max2500V instrument (Rigaku) with Cu-Kα radiation (*λ* = 1.5406 Å). The step was 0.02°, and the scan rate was 4°/min (scan range: 30–90°). Mössbauer spectroscopy was conducted with a Topologic 500 A spectrometer (Topologic Systems) and a proportional counter at room temperature. A 50 mCi ^57^Co (Rh) was employed as the γ-ray radioactive source in constant-acceleration mode. Isomer shifts of α-Fe were calibrated at room temperature. The hard XAS was measured on the 6D UNIST-PAL beamline in the Pohang Accelerator Laboratory (South Korea), and the data was fitted by Athena software. The XPS was measured on ESCALAB 250XI. The soft XAS was measured using the 4D PAL beamline in the Pohang Accelerator Laboratory (South Korea) in Ar atmosphere.

### Density functional theory calculations

All spin-polarized DFT calculations were performed by using the Dmol3 code in Materials Studio 2017. The exchange-correlation potential was based on the generalized gradient approximation with Perdew–Burke–Ernzerhof functional. The electron–core interactions were described by the DFT Semi-core Pseudopotential method, and the double numerical plus polarization (DNP) method was employed for basis sets with the real-space global orbital cutoff radius of 4.6 Å. To describe the van der Waals interactions, the Grimme (DFT-D2 correction) method was adopted. As well, linear synchronous transit/quadratic synchronous transit (LST/QST) methods were used to search for the transition state (TS). In this work, the model of BCC-Fe(110) surface was built in four atomic layers, and the bottom two layers of the atoms were fixed. During the calculation, the k-point sampling was set to 3 × 3 × 1 for integrating the Brillouin zones, and the convergence criterions of the energy, maximum force, and maximum displacement were 1 × 10^–5^ Ha, 0.002 Ha Å^−1^, and 0.005 Å, respectively.

## Supplementary information

Supplementary Information

## Data Availability

The data which were generated in this study are presented in the main text and Supplementary Information, and are available from the corresponding authors on reasonable request.
