# Supplementary material for: Achieving volatile potassium promoted ammonia synthesis via mechanochemistry
Source: Nat Commun. 2023 Apr 22;14:2319. doi: 10.1038/s41467-023-38050-2 (PMC10122650; doi:10.1038/s41467-023-38050-2)
Supplement: Supplementary file 1 — Supplementary Information [file 41467_2023_38050_MOESM1_ESM.pdf]

Supplementary Information

for

Achieving volatile potassium promoted ammonia synthesis via mechanochemistry

Jong-Hoon Kim, Tian-Yi Dai, Mihyun Yang, Jeong-Min Seo, Jae Seong Lee, Do Hyung Kweon, Xing-You Lang, Kyuwook Ihm, Tae Joo Shin, Gao-Feng Han, Qing Jiang and Jong-Beom Baek

Correspondence to: Jong-Beom Baek, jbbaek@unist.ac.kr; Qing Jiang, jiangq@jlu.edu.cn; Gao-Feng Han, gfhan@jlu.edu.cn

This Supplementary Information includes:

Supplementary Notes
Supplementary Figures 1–20
Supplementary Tables 1, 2
Supplementary References

Supplementary Notes

Study on crystalline grain.

We used the Scherrer equation[1] to estimate the grain size (τ) of the catalyst using equation (1).

$$\tau = \frac{K\lambda}{\beta\cos\theta} \quad (1)$$

where K is the shape factor, with a typical value of 0.9, λ is the Cu Kα wavelength (1.5406 Å), β is full width at half maximum (FWHM), and θ is the Bragg angle.

We used the (110) X-ray diffraction (XRD) peak to estimate the grain size of each catalyst. Each of the catalysts' (110) facet Bragg angles were 22.15°, 22.32°, 22.01°, and 22.34°, FWHMs were 2.689°, 0.768°, 3.318°, and 1.103°, and thus the grain sizes were 3.16 nm, 11.20 nm, 2.56 nm, and 7.75 nm, respectively, for FeKN*, FeK, FeN*, and Fe.

Calculation for bulk N content on each catalyst dependent on XRD measurement.

We also estimated the N concentration of bulk powder for FeKN* and FeN* using the following equations[2,3].

$$n\,\lambda = 2\,d_{hkl}\,\sin\theta \quad (2)$$

$$d_{hkl} = \frac{a_{\mathrm{Fe,N}}}{\sqrt{h^2+k^2+l^2}} \quad (3)$$

$$a_{\mathrm{Fe,N}} = a_{\mathrm{Fe}} + C \times (\text{at.\% of N}) \quad (4)$$

where, n is the diffraction order, d_{hkl} is the distance between the layers, h, k, l are Miller notations, a_{Fe} is the unit cell dimension of the α-Fe (2.8664 Å), $a_{\mathrm{Fe,N}}$ is the unit cell dimension of the N doped α-Fe and C is a conversion constant (0.0099 Å). The unit cell dimensions and the conversion constant of the FeK could be a bit different than that of α-Fe, but as can be seen in the XRD patterns of the 4 samples, they appear to be quite similar to each other. Therefore, we used the same value for all samples.

First, we chose the (110) XRD peak to calculate the d_{hkl} using equation (2). Because the sum of Miller notations (h, k, l) is the even number, the n should be 1. The Bragg angles of FeKN* and FeN* were 22.15°, and 22.01°, respectively. After determining the d_{hkl}, we calculated $a_{\mathrm{Fe,N}}$ for FeKN* and FeN* using equation (3). Finally, we used equation (4) to calculate the bulk atomic ratio of the absorbed N on FeKN* and FeN*, which were 2.32 at% and 4.08 at%, respectively.

Calculation for weighted ammonia

The weighted ammonia synthesis rate was calculated with equation (5). We considered both N_2 dissociation time and hydrogenation time to calculate the weighted ammonia synthesis rate.

$$v_{\mathrm{NH_3}} = \frac{v_{\mathrm{N}} \times v_{\mathrm{H_2}}}{v_{\mathrm{N}} + v_{\mathrm{H_2}}} \qquad (5)$$

$$v_{\mathrm{N}} = 2 \times v_{\mathrm{N_2}} \qquad (6)$$

where $v_{\mathrm{N_2}}$ is the nitrogen gas adsorption and dissociation rate, $v_{\mathrm{H_2}}$ is the hydrogenation rate, v_{N} is the nitrogen atom adsorption rate.

Density Functional Theory Calculations for reaction rate constant *k* and turnover frequency (TOF)

The reaction rate constant *k* is calculated by transition-state (TS) theory[4], where an activation energy (E_a) is required to overcome the energy barrier of RDS, and *k* is determined by:

$$k = \frac{k_{\mathrm{B}}T}{h} \exp\left(-\frac{E_{\mathrm{a}}}{k_{\mathrm{B}}T}\right) \qquad (7)$$

where E_a values of the hydrogenation processes are 1.46 eV for FeK and 1.65 eV for Fe, respectively (Supplementary Fig. 8).

The TOF (in s^{-1}) calculation is based on RDS model[5], which is represented by:

$$\mathrm{TOF} = k_1 \left(1 + \frac{p_{\mathrm{NH_3}}}{K_2 p_{\mathrm{H_2}}^{1.5}}\right)^{-2} \qquad (8)$$

where $p_{\mathrm{H_2}}$ and $p_{\mathrm{NH_3}}$ represent the partial pressures of H_2 and NH_3, respectively. k_1 is the reaction rate constant (a footnote is added for clarity), and K_2 is the equilibrium constant of the hydrogenation process ($N^* + \frac{3}{2}H_2(g) \rightleftharpoons NH_3(g) + *$), which is calculated by:

$$K_2 = \exp\left(-\frac{\Delta G_{\mathrm{NH_3}}}{k_{\mathrm{B}}T}\right) \qquad (9)$$

where $\Delta G_{\mathrm{NH_3}}$ is the formation energy of the NH_3 molecule, and the values are 0.70 eV for FeK and 0.88 eV for Fe, respectively (Supplementary Fig. 9).

As the initial $p_{\mathrm{H_2}}$ is 9 bar according to the experiment conditions, the $p_{\mathrm{NH_3}}$ can be represented as $\frac{2}{3}(9 - p_{\mathrm{H_2}})$. The E_{a} and $\Delta G_{\mathrm{NH_3}}$ values are taken from DFT calculations. In this case, the TOF values of Fe and FeK are depended on the partial pressures of H_2 and the surface temperature *T* in the ball-milling process.

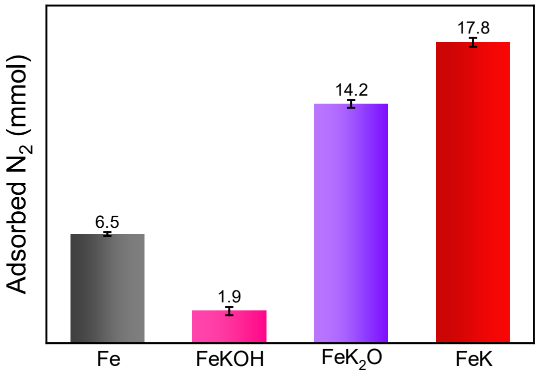

Supplementary Fig. 1 | Comparison of the amount of adsorbed N_2 with same catalyst weight, using different K promotors (K, K_2O, and KOH) at the same conditions [same loading ratio of K to Fe (2.5 at%), 45 °C] *via* mechanochemical process. For N_2 dissociation step, ball-milled for 11.5 h after charging N_2 (9 bar).

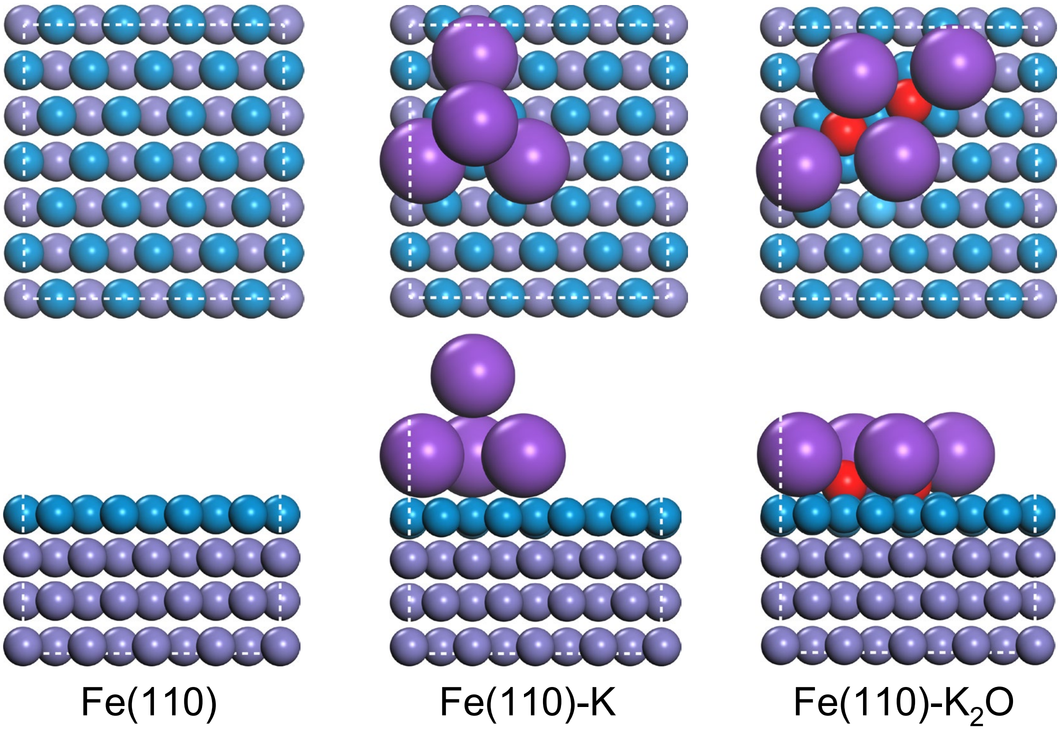

Supplementary Fig. 2 | Models for theoretical analysis. Geometric structural models (top and side views) of pure Fe(110) facet (left), and K (middle) and K_2O (right) on Fe(110) facet. The (110) facet is the lowest energy facet of α-Fe, namely, the most exposed Fe facet, we have chosen the models that K and K_2O are on the Fe(110) facet. Color code in the cartoon: purple, red, grey, and dark cyan are K, O, Fe, and surface Fe respectively.

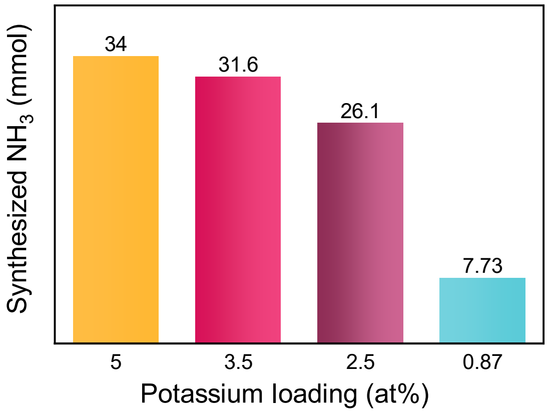

Supplementary Fig. 3 | Amount of synthesized NH_3 depending on K loading amount. The nitrogen dissociation was carried out at 450 r.p.m. under N_2 (9 bar) for 11.5 h and the hydrogenation was carried out at 500 r.p.m. under H_2 (9 bar) for 3 h.

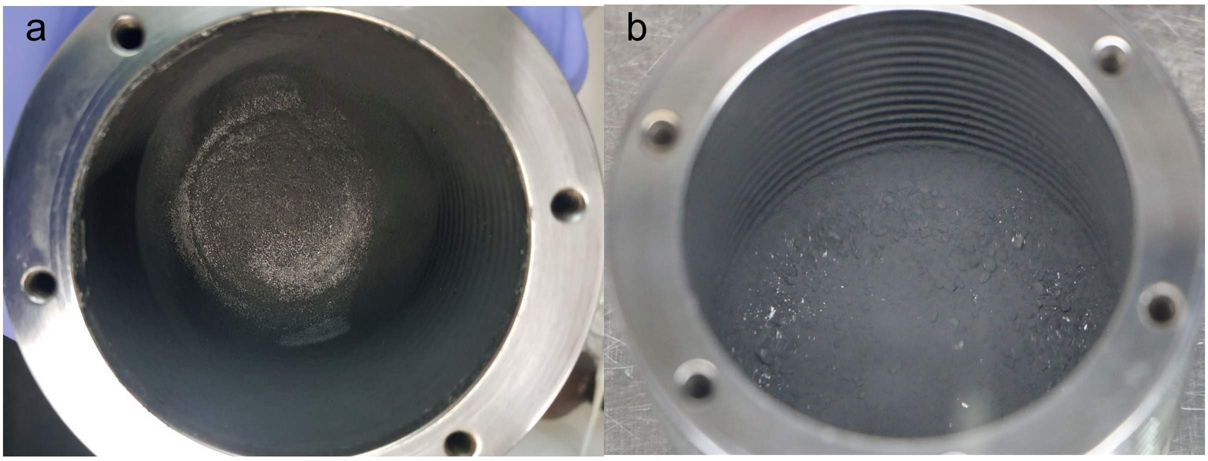

Supplementary Fig. 4 | The photo of the inside of the ball-mill container after hydrogenation. **a**, 5 at% K with Fe and **b**, 2.5 at% K with Fe. 5 at% K with Fe shows high agglomeration after ball-milling.

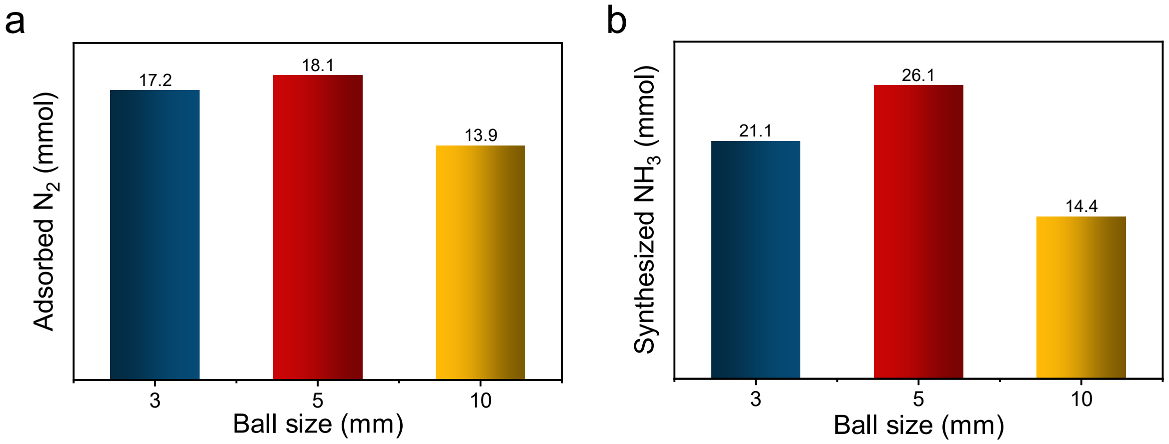

Supplementary Fig. 5 | a, Amount of adsorbed N_2 after nitrogenation. For each experiment with a specific ball size, the nitrogenation was carried out at the rotation speed of 450 r.p.m. for 11.5 h after charging N_2 (9 bars). **b**, Yields of synthesized ammonia after hydrogenation. The hydrogenation was conducted at the rotation speed of 500 r.p.m. for 3 h after charging H_2 (9 bars).

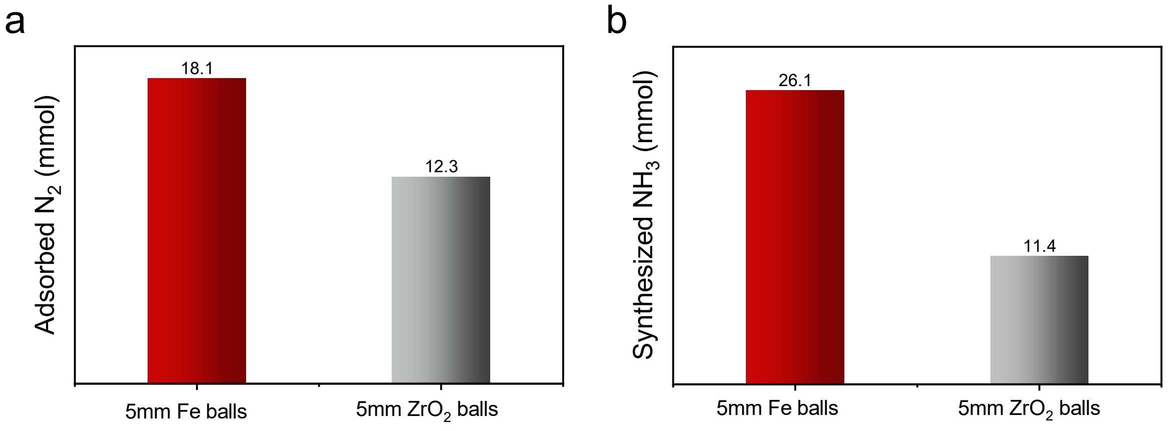

Supplementary Fig. 6 | Comparisons of activity with different ball types. **a**, Amount of adsorbed N_2 after nitrogenation. For Fe and ZrO_2 balls, the nitrogenation was carried out at the rotation speeds of 450 and 490 r.p.m., respectively, for 11.5 h after charging N_2 (9 bars). **b**, Yields of synthesized ammonia after hydrogenation. The hydrogenations for Fe and ZrO_2 balls were conducted at the rotation speeds of 500 and 540 r.p.m., respectively, for 3 h after charging H_2 (9 bars). Considering the difference in kinetic energy ($E_{\mathrm{K}} = \frac{1}{2} m \times v^2$) between different type balls, we used the same number of ZrO_2 balls and calculated the rotation speed required to maintain the same kinetic energy as the Fe balls.

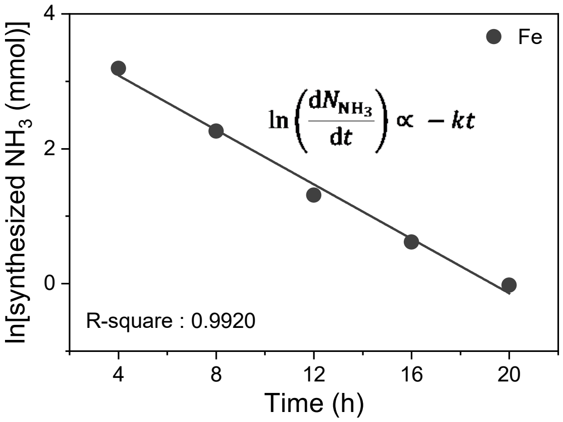

Supplementary Fig. 7 | Hydrogenation kinetics of Fe. Relation between the natural logarithm of synthesized NH_3 *vs.* time shows a linear dependence for pure Fe. Data were adapted from ref.[6]

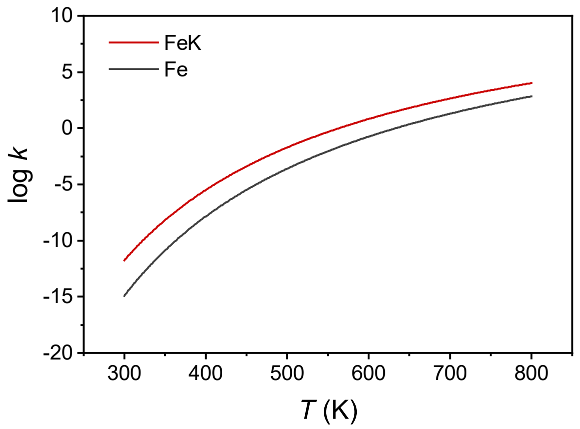

Supplementary Fig. 8 | Comparation of the reaction rate constant (*k*) between the FeK and Fe at different surface temperature.

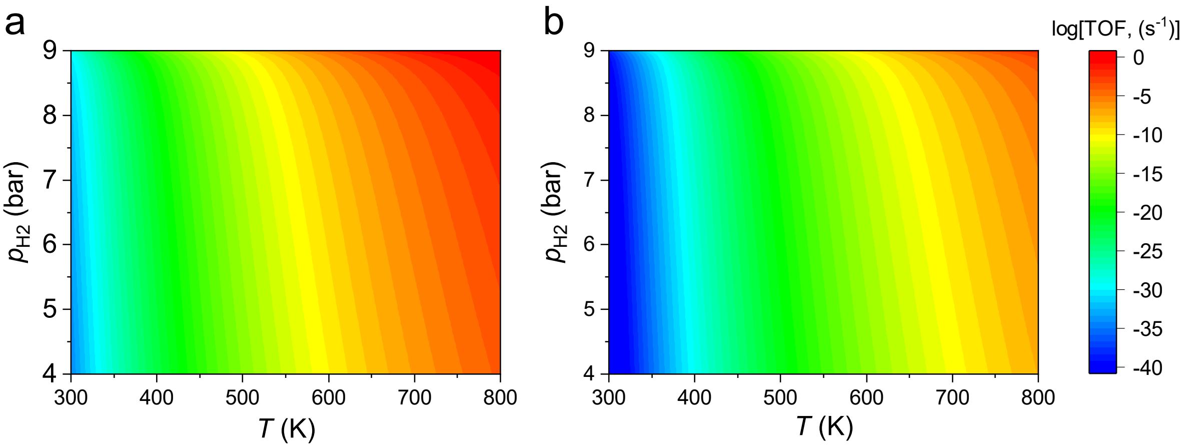

Supplementary Fig. 9 | 2D activity heatmap describing the turnover frequency (TOF) to NH_3 in hydrogenation process as a function of partial pressures of H_2 (p_{H_2}) and the surface temperature (*T*) on **a**, FeK and **b**, Fe.

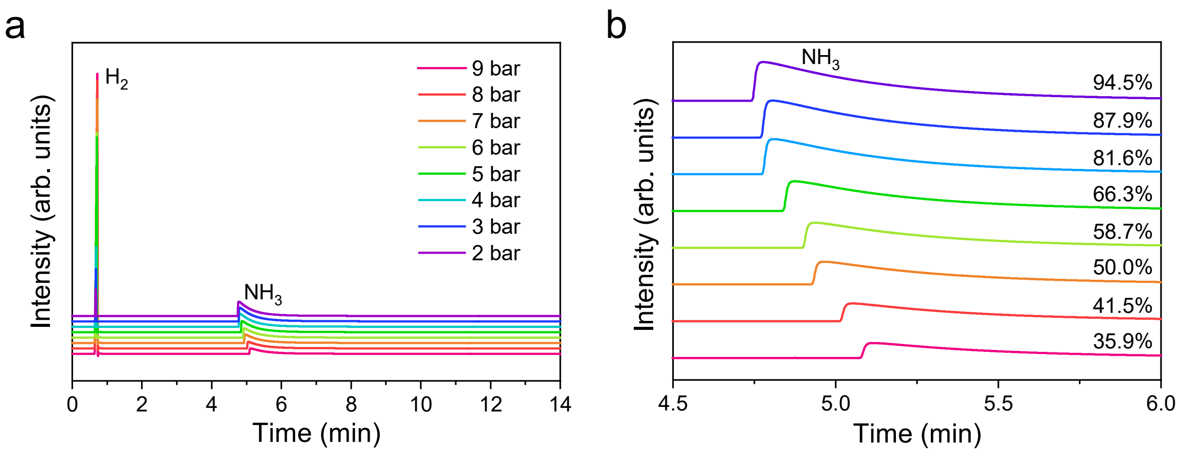

Supplementary Fig. 10 | Gas chromatography (GC) curves with respect to different hydrogen pressure. GC measurements were conducted after sequential reactions of nitrogen dissociation (N_2, 9 bar) for 18 h to form FeKN* and the hydrogenation at different H_2 pressure (2, 3, 4, 5, 6, 7, 8, or 9 bar) for 2 h each. **a**, The full range of GC curves. **b**, The magnified GC curves, showing NH_3 concentrations.

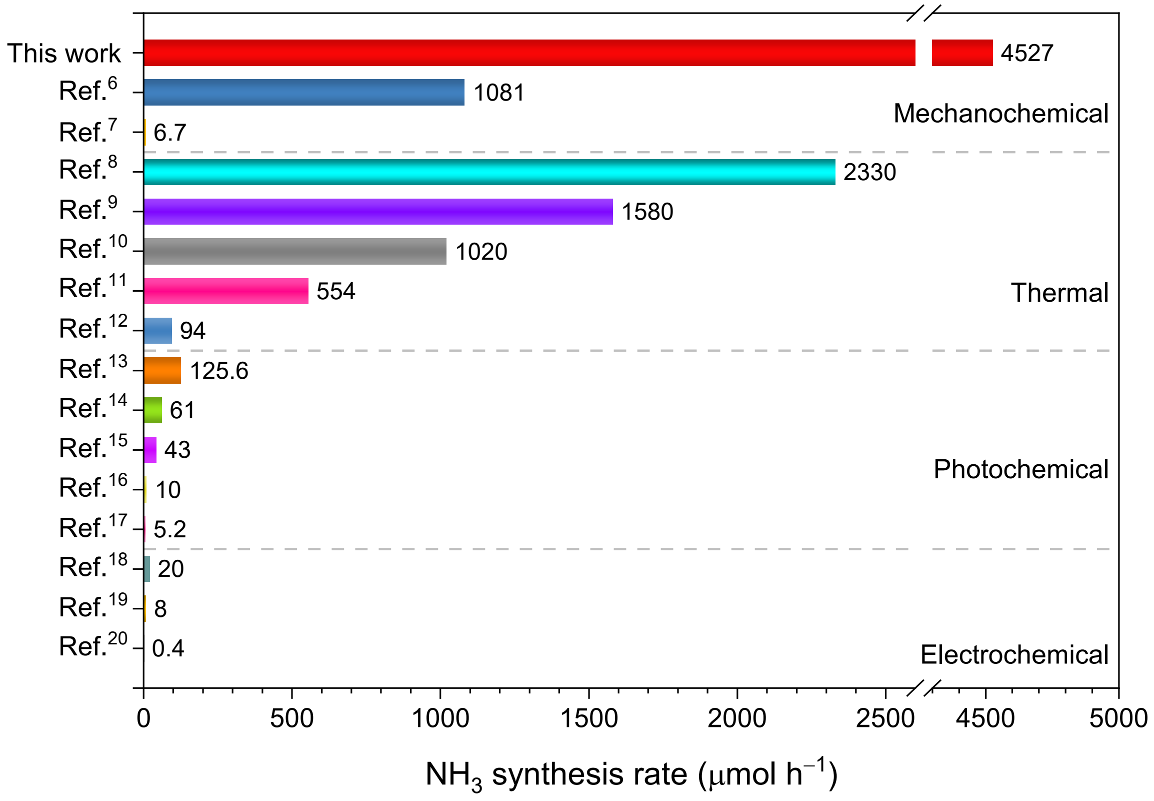

Supplementary Fig. 11 | Comparison of the NH_3 synthesis rate with those in recent works. The numbers in the y-axis are the work numbers presented in Supplementary Table 1.

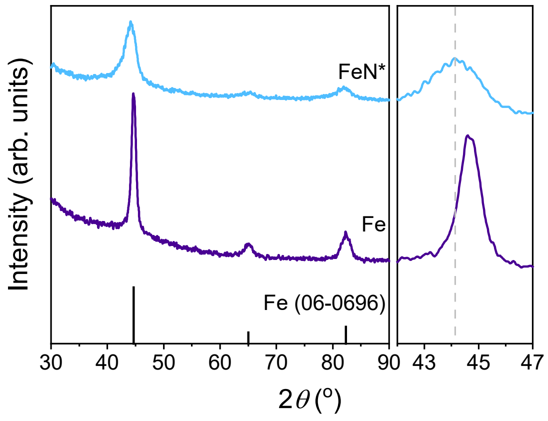

Supplementary Fig. 12 | XRD measurements of FeN* and Fe. After nitrogenation, the full width at half maximum became wider, and the peak was shifted to low angle.

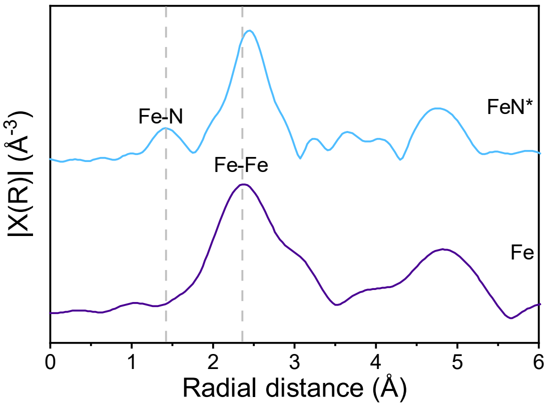

Supplementary Fig. 13 | Radial distribution function of FeN* and Fe. The radial distribution function was fitted using Fe K-edge EXAFS data.

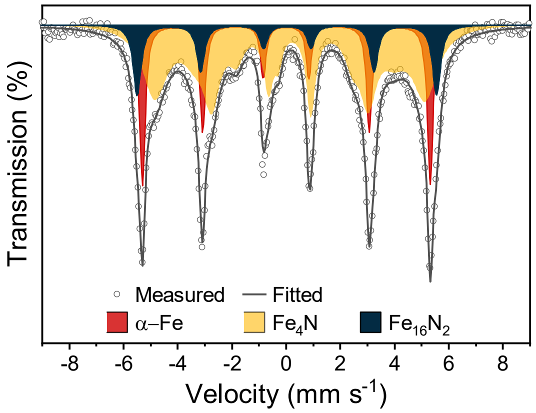

Supplementary Fig. 14 | Mössbauer spectra of FeN*. FeN* consisted of the nearly amorphous Fe_4N phase (62%) with broad peaks, crystalline $Fe_{16}N_2$ (14%), and crystalline α-Fe (23%) with sharp peaks.

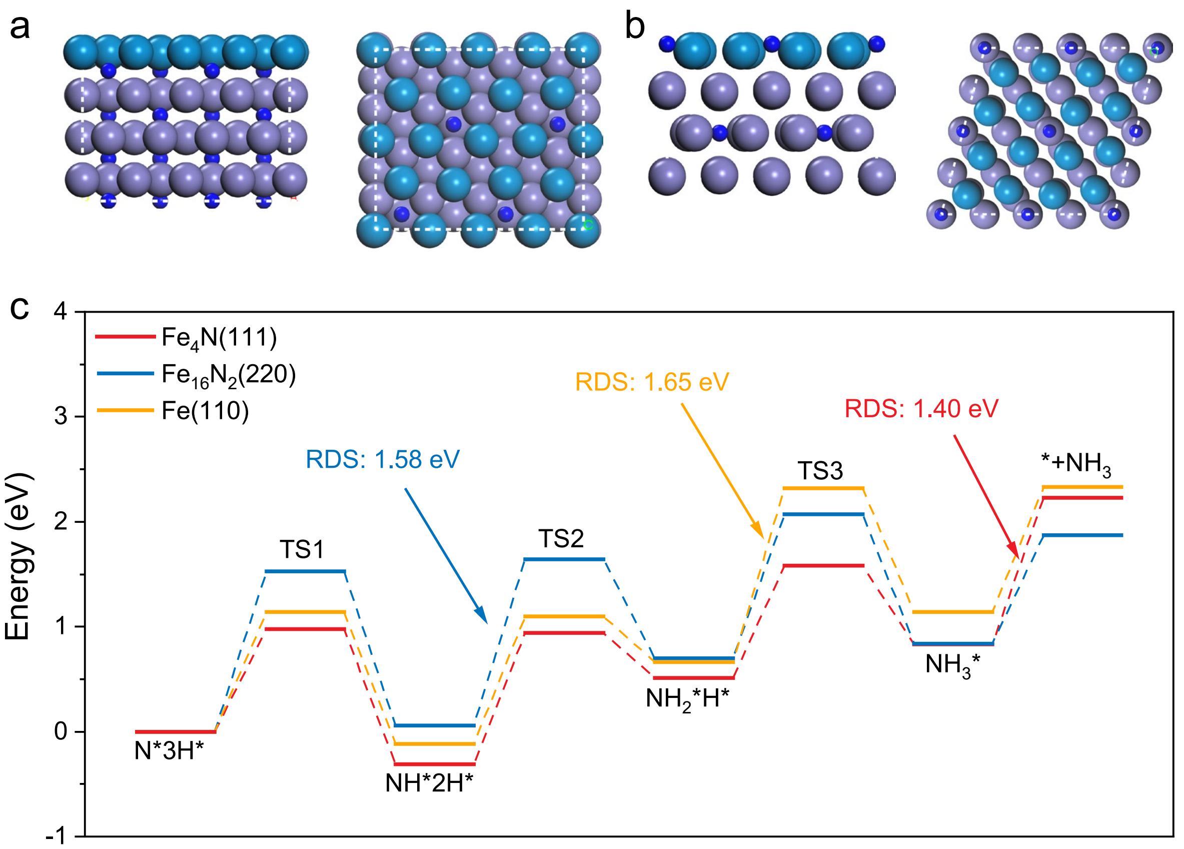

Supplementary Fig. 15 | Theoretical analysis for hydrogenation. DFT models: **a**, Fe_4N. **b**, $Fe_{16}N_2$. **c**, Energy profiles of hydrogenation for $Fe_4N(111)$, $Fe_{16}N_2(220)$, and Fe(110). Color code in the cartoon: blue, grey and dark cyan are N, Fe, and surface Fe, respectively.

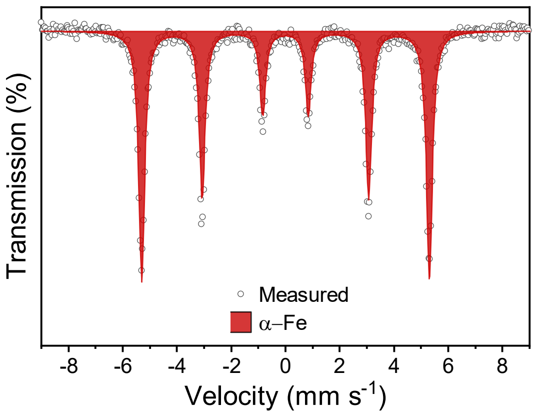

Supplementary Fig. 16 | Mössbauer spectra of regenerated FeK after the hydrogenation of FeKN* into NH_3. Compared with FeKN*, only the sextets of α-Fe can be identified. This implies all the nitrogen adsorbed during nitrogenation can be turned into NH_3 after hydrogenation.

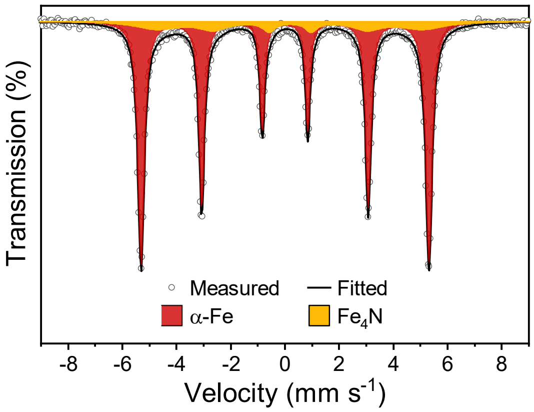

Supplementary Fig. 17 | Mössbauer spectra of regenerated Fe after the hydrogenation of FeN* into NH_3. Compared with FeKN*, low amount of Fe_4N was left. It suggests most of the nitrogen can be changed into NH_3 during hydrogenation.

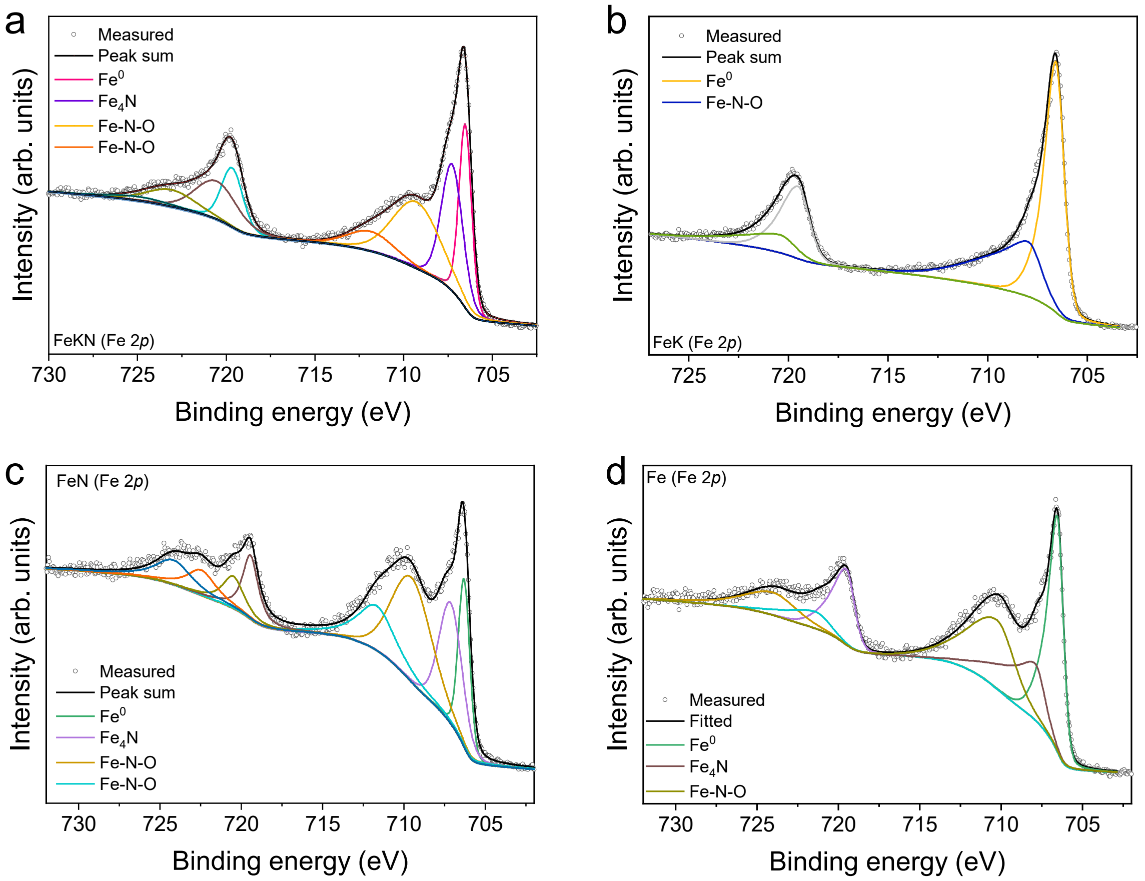

Supplementary Fig. 18 | X-ray photoelectron spectroscopy of FeKN*, FeK, FeN*, and Fe. High resolution Fe 2*p* spectra: **a**, FeKN* powders; **b**, FeK powders; **c**, FeN* powders; **d**, Fe powders.

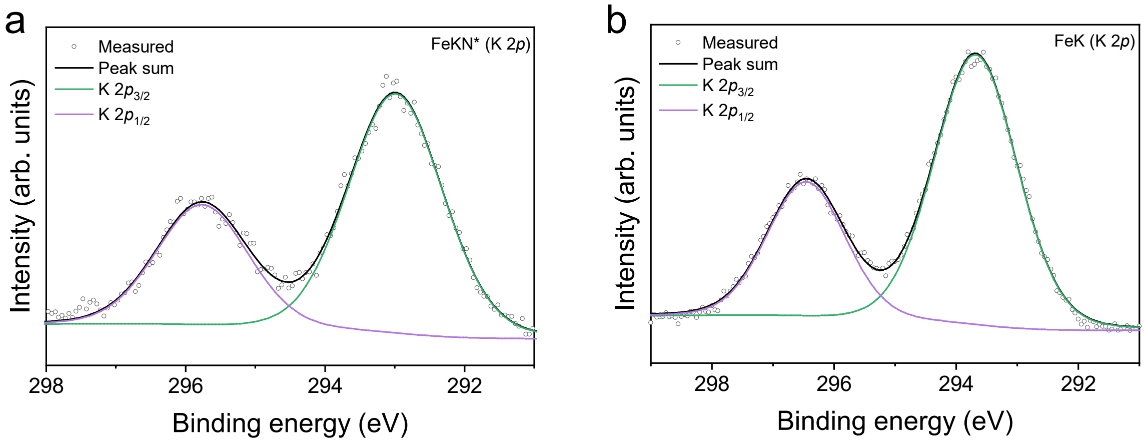

Supplementary Fig. 19 | X-ray photoelectron spectroscopies of FeKN* and FeK. High-resolution K 2*p* spectra: **a**, FeKN* powders; **b**, FeK powders.

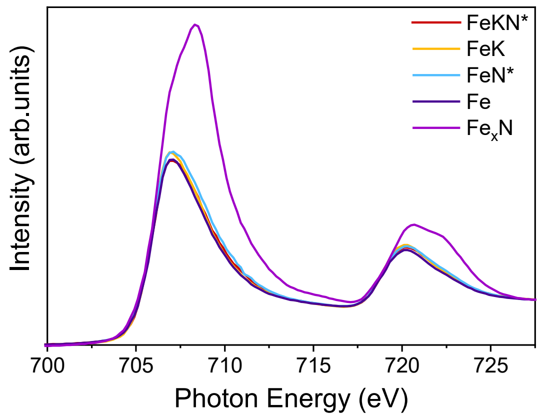

Supplementary Fig. 20 | Soft XAS spectra for Fe L-edge of FeKN*, FeK, FeN*, Fe, and commercial Fe_xN (x = 2–4). There is no appreciable difference among FeKN*, FeK, FeN*, and Fe. However, FeKN*, FeK, FeN*, and Fe showed lower energy than commercial Fe_xN (x = 2–4). This indicates the bonding between Fe and N* in FeKN* and FeN* is weak.

Supplementary Table 1 | Comparison of NH_3 production rate with those in recent work

Method	Work No.	Catalyst	Reaction Cond.	Product phase	Production per h (μmol h^{-1})	Production per g and h (μmol g^{-1} h^{-1})
Mechano-chemical	This work	FeK	45 °C, 1 bar	Gas	[6704 (υ_N)][a]<br>[13945 (υ_{H_2})][b]<br>[4527(υ_{NH_3})][c]	281<br>585<br>190
Mechano-chemical	ref.[6]	Fe	45 °C, 1 bar	Gas	[1315 (υ_N)][d]<br>[6073 (υ_{H_2})][e]<br>[1081(υ_{NH_3})][f]	55<br>253<br>45
Mechano-chemical	ref.[7]	Fe	25 °C, 1 bar	Electrolyte	6.7	-
Thermal	ref.[8]	Ru/Ba-$Ca(NH_2)_2$	300 °C, 9 bar	Gas	2330	23300
Thermal	ref.[9]	Ru/ $Ca(NH_2)_2$	300 °C, 8 bar	Gas	1580	15800
Thermal	ref.[10]	Ba_2RuH_6/ MgO	300 °C, 10 bar	Gas	1020	34000
Thermal	ref.[11]	Ni/LaN	400 °C, 1 bar	Gas	554	5543
Thermal	ref.[12]	Ni–BaH_2	350 °C, 1 bar	Gas	94	3125
Photo-chemical	ref.[13]	B-N-C	ambient	Electrolyte	125.6	313.9
Photo-chemical	ref.[14]	BiO	25 °C	Electrolyte	61	1226
Photo-chemical	ref.[15]	Mn-WO_3	ambient	Electrolyte	43	425
Photo-chemical	ref.[16]	Fe-Doped BiOCl	ambient	Electrolyte	10	1022
Photo-	ref.[17]	BiOBr	25 °C	Electrolyte	5.2	104.2

chemical						
Electro-chemical	ref.[18]	Fe_2O_3@MoS_2	ambient	Electrolyte	20	6597
Electro-chemical	ref.[19]	F-Fe: TiO_2	ambient	Electrolyte	8	1639
Electro-chemical	ref.[20]	PdPb/C	ambient	Electrolyte	0.4	2216

[a] v_N is the nitrogen atom adsorption rate (The rotation speed of nitrogenation for FeK was 400 r.p.m.).

[b] v_{H_2} is the hydrogenation rate (The rotation speed of hydrogenation for FeK was 500 r.p.m.).

[c, f] Weighted ammonia synthesis rate (v_{NH_3}) which was calculated by supplementary text equation (5).

[d] v_N is the nitrogen atom adsorption rate (The rotation speed of nitrogenation for Fe was 450 r.p.m.).

[e] v_{H_2} is the hydrogenation rate (The rotation speed of nitrogenation for Fe was 500 r.p.m.).

Supplementary Table 2 | Comparison of NH_3 synthesis rate depending on energy consumed with the results in previous work

**Work**	**Catalyst**	**Production per energy (mmol kWh^{-1})**
This work	FeK	[190.3 (v_N)][a]<br>[202.1 (v_{H_2})][b]<br>[98.0(v_{NH_3})][c]
Previous work[6]	Fe	[26.7 (v_N)][d]<br>[87.7 (v_{H_2})][e]<br>[20.5(v_{NH_3})][f]

[a] v_N is the nitrogen atom adsorption rate (The rotation speed of nitrogenation for FeK was 400 r.p.m.).

[b] v_{H_2} is the hydrogenation rate (The rotation speed of hydrogenation for FeK was 500 r.p.m.).

[c, f] Weighted ammonia synthesis rate (v_{NH_3}) which was calculated by supplementary text equation (5).

[d] v_N is the nitrogen atom adsorption rate (The rotation speed of nitrogenation for Fe was 450 r.p.m.).

[e] v_{H_2} is the hydrogenation rate (The rotation speed of nitrogenation for Fe was 500 r.p.m.).

Supplementary References

1. Authier, A. *Dynamical Theory of X-Ray Diffraction*. (Oxford University Press, 2001).
2. Bragg, W. H. & Bragg, W. L. The reflection of X-rays by crystals. *Proc. R. Soc. A* **88**, 428–438 (1913).
3. Jack, D. H. & Jack, K. H. Invited review: Carbides and nitrides in steel. *Mater. Sci. Eng.* **11**, 1–27 (1973).
4. Singh, A. R. *et al.* Computational Design of Active Site Structures with Improved Transition-State Scaling for Ammonia Synthesis. *ACS Catal.* **8**, 4017–4024 (2018).
5. Kozuch, S. & Shaik, S. A combined kinetic-quantum mechanical model for assessment of catalytic cycles: Application to cross-coupling and Heck reactions. *J. Am. Chem. Soc.* **128**, 3355–3365 (2006).
6. Han, G. F. *et al.* Mechanochemistry for ammonia synthesis under mild conditions. *Nat. Nanotechnol.* **16**, 325–330 (2021).
7. He, C. *et al.* Mechanochemical Synthesis of Ammonia Employing H_2O as the Proton Source Under Room Temperature and Atmospheric Pressure. *ACS Sustain. Chem. Eng.* **10**, 746–755 (2022).
8. Kitano, M. *et al.* Self-organized Ruthenium-Barium Core-Shell Nanoparticles on a Mesoporous Calcium Amide Matrix for Efficient Low-Temperature Ammonia Synthesis. *Angew. Chem. Int. Ed.* **57**, 2648–2652 (2018).
9. Inoue, Y. *et al.* Efficient and Stable Ammonia Synthesis by Self-Organized Flat Ru Nanoparticles on Calcium Amide. *ACS Catal.* **6**, 7577–7584 (2016).
10. Wang, Q. *et al.* Ternary ruthenium complex hydrides for ammonia synthesis via the associative mechanism. *Nat. Catal.* **4**, 959–967 (2021).
11. Ye, T. N. *et al.* Vacancy-enabled N_2 activation for ammonia synthesis on an Ni-loaded catalyst. *Nature* **583**, 391–395 (2020).
12. Gao, W. *et al.* Production of ammonia *via* a chemical looping process based on metal imides as nitrogen carriers. *Nat. Energy* **3**, 1067–1075 (2018).
13. Wang, W. *et al.* Formation of B-N-C Coordination to Stabilize the Exposed Active Nitrogen Atoms in g-C_3N_4 for Dramatically Enhanced Photocatalytic Ammonia Synthesis Performance. *Small* **16**, 1906880 (2020).
14. Sun, S. *et al.* Efficient photocatalytic reduction of dinitrogen to ammonia on bismuth monoxide quantum dots. *J. Mater. Chem. A* **5**, 201–209 (2017).
15. Zhang, Y. *et al.* Dual-Metal Sites Boosting Polarization of Nitrogen Molecules for Efficient Nitrogen Photofixation. *Adv. Sci.* **8**, 2100302 (2021).
16. Zhang, N. *et al.* Fe-Doped BiOCl Nanosheets with Light-Switchable Oxygen Vacancies for Photocatalytic Nitrogen Fixation. *ACS Appl. Energy Mater.* **2**, 8394–8398 (2019).
17. Li, H., Shang, J., Ai, Z. & Zhang, L. Efficient Visible Light Nitrogen Fixation with BiOBr Nanosheets of Oxygen Vacancies on the Exposed {001} Facets. *J. Am. Chem. Soc.* **137**, 6393–6399 (2015).
18. Ma, C. *et al.* MOF-derived Fe_2O_3@MoS_2: An efficient electrocatalyst for ammonia synthesis under mild conditions. *J. Chem. Eng.* **430**, 132694 (2022).
19. Song, G. *et al.* High-spin state Fe(III) doped TiO_2 for electrocatalytic nitrogen fixation induced by surface F modification. *Appl. Catal. B Environ.* **301**, 120809 (2022).
20. Zhao, H. *et al.* High-performance nitrogen electroreduction at low overpotential by introducing Pb to Pd nanosponges. *Appl. Catal. B Environ.* **265**, 118481 (2020).
